# Higher-level functional capacity and incident long-term care insurance certification: a competing risks analysis

**DOI:** 10.3389/fpubh.2026.1950765

**Published:** 2026-10-06

**Authors:** Meiling Qian, Jinrui Zhang, Shuanghong Li, Maiko Shigeeda, Haotian Gao, Ruifeng Zhao, Mengxuan Wang, Dandan Jiao, Yuko Sawada, Akihiro Kakuda, Hiroshi Kinoshita, Rika Okumura, Yuriko Kawasaki, Tokie Anme

**Affiliations:** 1 Graduate School of Comprehensive Human Sciences, University of Tsukuba, Tsukuba, Japan; 2 Department of Nursing, The First Affiliated Hospital and College of Clinical Medicine, Henan University of Science and Technology, Luoyang, Henan, China; 3 Department of Physical Therapy, Morinomiya University of Medical Sciences, Osaka, Japan; 4 Department of Public Welfare, Tobishima, Japan; 5 Faculty of Medicine, University of Tsukuba, Tsukuba, Ibaraki, Japan

**Keywords:** ageing, cohort study, competing risks, higher-level functional capacity, long-term care insurance, social role

## Abstract

**Background:**

Higher-level functional capacity (HLFC) may capture functional vulnerability beyond basic activities of daily living in older adults. However, the association of baseline HLFC with subsequent incident long-term care insurance (LTCI) certification remains unclear when death is considered a competing event.

**Methods:**

This prospective cohort study included 688 community-dwelling Japanese adults aged 65 years or older who participated in the 2017 wave of the Community Empowerment and Care for Well-being and Healthy Longevity Study. HLFC was assessed once at baseline using the Tokyo Metropolitan Institute of Gerontology Index of Competence (TMIG-IC), including the total score and the formal subdomain scores for Instrumental Self-Maintenance, Intellectual Activity, and Social Role. Participants were followed for 8 years. Incident LTCI certification at Support Level 1 or higher was analyzed using Fine–Gray competing-risk regression, with death before certification treated as a competing event. The fully adjusted Fine–Gray model included age group, sex, living arrangement, physical activity, smoking status, alcohol consumption, and disease status.

**Results:**

Among 688 complete-case participants, 145 developed incident LTCI certification and 79 died before certification. In the fully adjusted model, higher HLFC total score was associated with a lower cumulative incidence of incident LTCI certification (sHR per 1-point increase, 0.941; 95% CI, 0.891–0.994; *p* = 0.029). Higher Social Role score was also associated with a lower cumulative incidence (sHR, 0.803; 95% CI, 0.702–0.918; *p* = 0.001), whereas the continuous per-point Instrumental Self-Maintenance and Intellectual Activity estimates included the null after full adjustment. Multiple-imputation and lagged-event analyses generally retained the direction and broad magnitude of the complete-case estimates. Exposure-by-log(time) diagnostics indicated that the Fine-Gray associations varied over follow-up. Estimates also varied under alternative age specifications, with particularly marked attenuation of the total-score association.

**Conclusion:**

Higher baseline HLFC total and Social Role scores were associated with a lower subsequent cumulative incidence of incident LTCI certification in the prespecified age-group-adjusted analysis. The total-score association was substantially attenuated under alternative age adjustments, while the magnitude of the Social Role association also varied.

## 1 Introduction

Japan’s long-term care insurance (LTCI) system provides formal support for older adults who require assistance with daily living (1–3). Incident LTCI certification is widely used as an outcome in Japanese gerontological and epidemiologic research (4–6). With population ageing and increasing long-term care needs, identifying functional characteristics associated with subsequent LTCI certification is important for understanding later-life care dependency and for long-term care planning (3, 7, 8). Functional limitations develop across multiple levels of competence, and measures based only on basic activities of daily living (ADL) may be less sensitive to differences among older adults who remain largely independent in the community (9, 10).

Higher-level functional capacity (HLFC) describes abilities beyond basic ADL that are required for independent community living. Based on Lawton’s model of competence, the Tokyo Metropolitan Institute of Gerontology Index of Competence (TMIG-IC) assesses HLFC through the formal subdomains Instrumental Self-Maintenance, Intellectual Activity, and Social Role (11, 12). Instrumental self-maintenance reflects practical independence in community living, intellectual activity reflects information-related and cognitively demanding activities, and social role reflects interpersonal and community-related functioning. These dimensions extend beyond basic self-care and allow differences in later-life functioning to be characterized among older adults who may otherwise appear independent on conventional ADL measures (13).

Previous longitudinal studies have linked poorer higher-level functional capacity and unfavorable TMIG-IC trajectories with functional decline, mortality, and greater medical or long-term care use (13–16). Studies using repeated TMIG-IC measurements have also shown that the three subdomains may follow different age-related patterns over time (13, 17). In addition, baseline TMIG-IC scores have been examined in relation to subsequent LTCI certification (18). Other Japanese cohort studies have identified cognitive, physical, social, and multidimensional baseline characteristics associated with subsequent LTCI certification (19–22). However, evidence remains limited on these associations over longer follow-up when death before certification is explicitly treated as a competing event.

This competing event is particularly relevant in older populations because individuals who die before LTCI certification can no longer experience the outcome of interest. Analyses that do not explicitly account for competing death address a different risk process from analyses focused on the cumulative incidence of LTCI certification in the presence of mortality (23, 24). A competing-risk framework therefore provides an appropriate approach for examining subsequent LTCI certification during long-term follow-up (23, 25).

The TMIG-IC total score provides an overall measure of HLFC, whereas its three subdomains represent different aspects of later-life competence (12). Although baseline TMIG-IC scores have previously been examined in relation to subsequent LTCI certification (18), less is known about how the individual subdomains relate to certification over longer follow-up when competing mortality is explicitly accounted for.

Accordingly, this study examined the associations of baseline HLFC total and subdomain scores with subsequent incident LTCI certification over 8 years of follow-up among community-dwelling older Japanese adults, while accounting for death as a competing event using Fine-Gray models.

## 2 Methods

### 2.1 Design and participants

This study used data from the Community Empowerment and Care for Well-being and Healthy Longevity (CEC) Study, an ongoing population-based cohort conducted in a rural village in Aichi Prefecture, Japan. The CEC Study was launched in 1991 to investigate social, behavioral, and health-related factors associated with aging, and follow-up surveys have been conducted every 3 years. At each wave, self-administered questionnaires are distributed to community-dwelling residents aged 65 years or older, and municipal records provide LTCI certification and vital-status information. In the 2017 wave, 1,301 residents were identified from the municipal registry and 1,118 returned valid questionnaires (response rate, 85.9%). After 135 participants with prevalent LTCI certification were excluded, 983 were eligible for the incident-outcome analysis. A further 295 were excluded because of missing data required for the primary analysis, leaving 688 participants in the complete-case analytic sample (Figure 1). Participants were followed from April 1, 2017, to March 31, 2025.

**Figure 1:**
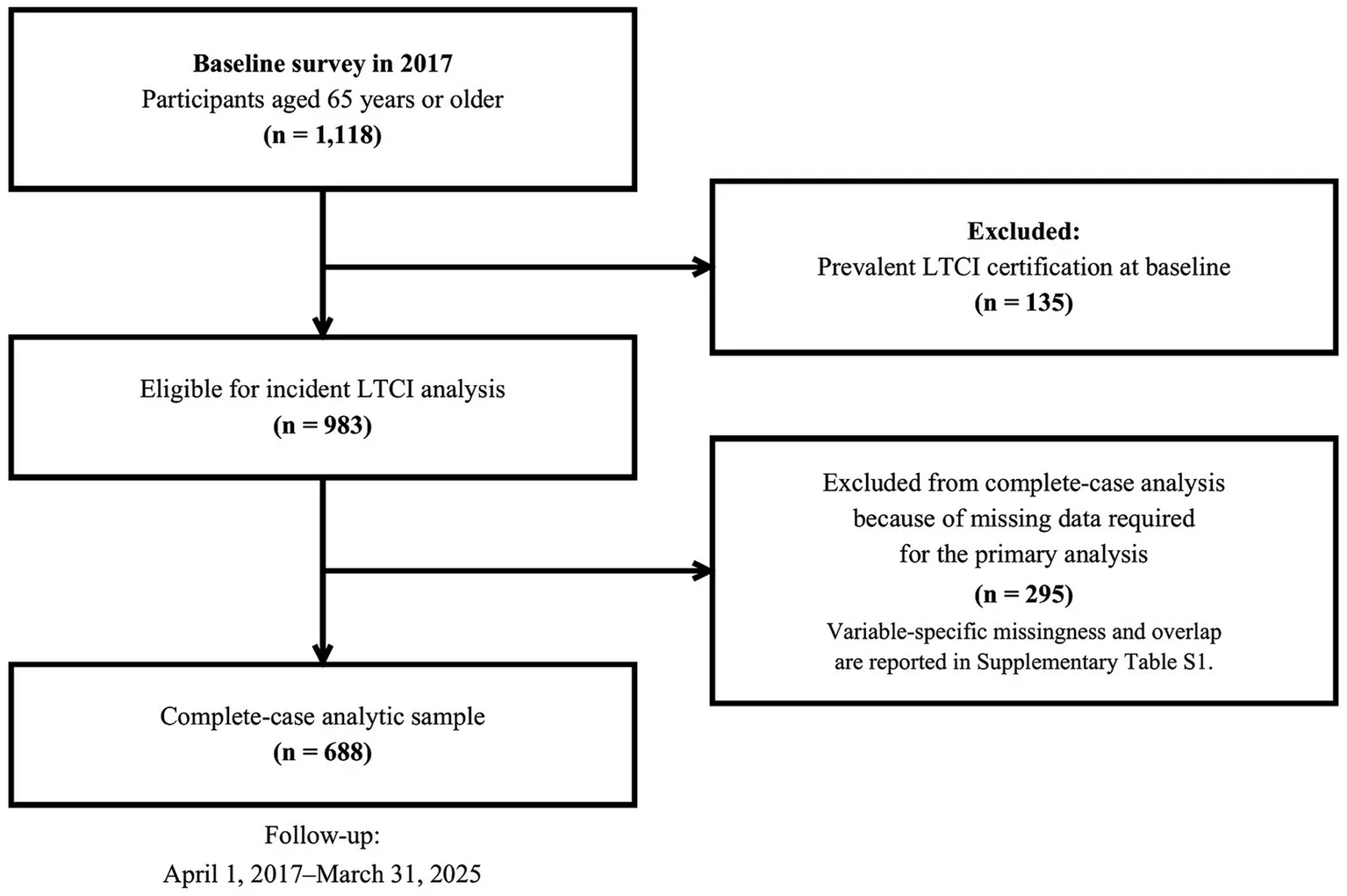
Participant flow.

### 2.2 Assessment of higher-level functional capacity

HLFC was assessed once at baseline using the TMIG-IC, a validated 13-item scale of higher-level competence among older adults (12). The conceptual basis derives from Lawton’s model of competence, which describes functional abilities beyond basic activities of daily living (11). Each item was scored as 1 (yes) or 0 (no), yielding a total score from 0 to 13, with higher scores indicating higher HLFC. The three formal subdomains are Instrumental Self-Maintenance (five items), Intellectual Activity (four items), and Social Role (four items). Their items are shown in Supplementary Table S9. The TMIG-IC has demonstrated acceptable reliability and validity among older Japanese adults (12). The total and subdomain scores were treated as continuous variables in the primary models.

### 2.3 Assessment of incident LTCI certification

The outcome was incident LTCI certification, defined as a new certification under Japan’s LTCI system at Support Level 1 or higher, including Support Levels 1–2 and Care Levels 1–5 (2, 26). Certification is observed only after an application and a standardized assessment and certification process; it is not automatically recorded when health or functioning deteriorates. Following an application, trained municipal staff conduct an assessment using a structured questionnaire covering physical, cognitive, and social functions, and the municipality requests a written opinion from the applicant’s attending physician. A computer-based first-stage judgment is followed by review by the municipal Long-Term Care Certification Committee, comprising health, medical, and welfare experts (2, 27). Municipal administrative records provided the date of first LTCI certification.

### 2.4 Assessment of covariates

Several self-reported covariates were considered as potential confounders, including age group (65–74 years or 75 years or older), sex (male or female), living arrangement (alone or with others), physical activity (active or inactive, defined as exercising at least once per week), alcohol consumption (daily or non-daily), smoking status (never, former, or current), and disease status. Disease status was assessed using a self-reported checklist of physician-diagnosed conditions for which participants were receiving treatment or had residual sequelae. Participants were classified as having disease if they reported at least one of the following: hypertension, stroke, heart disease, diabetes mellitus, dyslipidemia, respiratory disease, gastrointestinal or hepatobiliary disease, renal or prostate disease, musculoskeletal disease, external injury, cancer, hematological or immune disease, depression, dementia, Parkinson’s disease, eye disease, or ear disease.

### 2.5 Statistical analysis

Baseline characteristics were summarized as mean ± standard deviation (SD) for continuous variables and as counts and percentages for categorical variables, overall and by follow-up outcome. Associations of baseline HLFC, assessed using the TMIG-IC total and subdomain scores, with incident LTCI certification were estimated using Fine–Gray competing-risk regression, with death before LTCI certification treated as a competing event (25). The TMIG-IC total score and each of the three subdomain scores were examined separately. Model 1 was unadjusted. Model 2 was adjusted for age group and sex. Model 3 was additionally adjusted for living arrangement, physical activity, smoking status, alcohol consumption, and disease status. Subdistribution hazard ratio (sHR) and 95% confidence interval (CI) were reported. The complete-case analysis was the primary analysis.

Among the 983 eligible participants, variable-specific missingness and overlap of missing data were described. Baseline characteristics of participants included in and excluded from the complete-case analysis were compared using standardized mean differences without hypothesis testing. Multiple imputation was conducted as a sensitivity analysis under a missing-at-random working assumption. Fifty imputations were generated over 30 iterations. The 13 TMIG-IC items were imputed at the item level, after which the total and three subdomain scores were reconstructed deterministically. Follow-up time, LTCI event status, and competing-death status were not imputed but were included as predictors in the imputation models. Binary variables were imputed using logistic regression, and three-category smoking status was imputed using multinomial logistic regression.

For each TMIG-IC measure, the proportional subdistribution hazards assumption in Model 3 was assessed using an exposure-by-log (follow-up time) interaction. When non-proportionality was detected, time-specific sHRs were examined to describe changes in the association over follow-up. Score distributions and quadratic terms were examined to assess potential nonlinearity. Because Instrumental Self-Maintenance showed substantial clustering at the maximum score, it was additionally examined as <5 versus 5 and in tie-preserving score groups of 0–3, 4, and 5.

For descriptive presentation of Social Role, cumulative incidence functions for incident LTCI certification were estimated for baseline score groups of 0–2, 3, and 4, with death treated as a competing event. Identical integer scores were not divided across groups. Pointwise 95% confidence intervals were estimated using 2,000 bootstrap resamples.

Additional sensitivity analyses excluded incident LTCI certification occurring within 183 or 365 days after baseline and excluded deaths occurring within 365 days. Subdomain associations were also estimated per 1-SD higher baseline score. To examine the subdomains jointly, all three were entered simultaneously in Model 3. Standardized coefficients were compared using covariance-based pairwise Wald tests. Spearman rank correlations, tolerance, and variance inflation factors were calculated to assess correlations and multicollinearity among the subdomains.

Cause-specific Cox regression models were additionally fitted separately for incident LTCI certification and death before LTCI certification, with the complementary event treated as censored. Proportional hazards diagnostics were assessed for these models, and time-specific hazard ratios were examined for the incident-LTCI endpoint when non-proportionality was detected. As an age-specification sensitivity analysis, the primary age-group variable was replaced with continuous age in years. To assess potential nonlinearity, additional Fine–Gray models included centered continuous age and a centered quadratic age term. These models were treated as diagnostic sensitivity analyses and did not replace the prespecified age-group-adjusted primary model.

Moving-date records were used to verify censoring. Participants who moved out of the municipality without prior LTCI certification or death were censored at the date of moving, whereas participants remaining event-free were administratively censored on March 31, 2025. All analyses were performed using R version 4.5.2, with two-sided *p* < 0.05 considered statistically significant.

## 3 Results

### 3.1 Baseline characteristics

Of 1,118 baseline participants aged 65 years or older, 135 with prevalent LTCI certification were excluded, leaving 983 eligible participants. After exclusion of 295 participants with missing data required for the primary analysis, 688 participants comprised the complete-case sample (Figure 1). During follow-up, 145 participants developed incident LTCI certification, 79 died before certification, and 464 remained without LTCI certification.

Among the 688 participants, 37.2% were aged 75 years or older, 50.9% were female, 60.3% were physically active, and 84.7% were classified as having disease. The mean HLFC total score was 10.9 ± 2.6; mean subdomain scores were 4.6 ± 1.1 for instrumental self-maintenance, 3.3 ± 1.0 for intellectual activity, and 3.0 ± 1.2 for social role (Table 1). Participants who subsequently received LTCI certification or died before certification generally had lower baseline HLFC scores than those who remained without certification. Compared with included participants, excluded participants were older and differed particularly in alcohol consumption, with smaller differences in sex, physical activity, and smoking (Supplementary Table S1).

**Table 1:** Baseline characteristics of participants according to incident LTCI certification and death status.

Variable	Total (*n* = 688)	Incident LTCI (*n* = 145)	No LTCI (*n* = 464)	Death before LTCI (*n* = 79)
Age group, *n* (%)				
65–74 years	432 (62.8)	50 (34.5)	344 (74.1)	38 (48.1)
≥75 years	256 (37.2)	95 (65.5)	120 (25.9)	41 (51.9)
Sex, *n* (%)				
Female	350 (50.9)	78 (53.8)	249 (53.7)	23 (29.1)
Male	338 (49.1)	67 (46.2)	215 (46.3)	56 (70.9)
Disease status, *n* (%)				
Having disease	583 (84.7)	127 (87.6)	381 (82.1)	75 (94.9)
Without disease	105 (15.3)	18 (12.4)	83 (17.9)	4 (5.1)
Physical activity, *n* (%)				
Active	415 (60.3)	73 (50.3)	297 (64.0)	45 (57.0)
Inactive	273 (39.7)	72 (49.7)	167 (36.0)	34 (43.0)
Alcohol consumption, *n* (%)				
Non-daily	425 (61.8)	106 (73.1)	275 (59.3)	44 (55.7)
Daily	263 (38.2)	39 (26.9)	189 (40.7)	35 (44.3)
Smoking status, *n* (%)				
Current	83 (12.1)	17 (11.7)	49 (10.6)	17 (21.5)
Former	180 (26.2)	35 (24.1)	118 (25.4)	27 (34.2)
Never	425 (61.8)	93 (64.1)	297 (64.0)	35 (44.3)
Living arrangement, *n* (%)				
Alone	41 (6.0)	7 (4.8)	31 (6.7)	3 (3.8)
With others	647 (94.0)	138 (95.2)	433 (93.3)	76 (96.2)
HLFC score, mean ± SD				
Total	10.9 ± 2.6	10.0 ± 3.2	11.4 ± 2.1	9.6 ± 3.4
Instrumental Self-Maintenance	4.6 ± 1.1	4.3 ± 1.3	4.8 ± 0.8	4.1 ± 1.7
Intellectual Activity	3.3 ± 1.0	3.0 ± 1.2	3.4 ± 0.8	2.9 ± 1.2
Social Role	3.0 ± 1.2	2.7 ± 1.4	3.2 ± 1.1	2.6 ± 1.3

Values are expressed as *n* (%) or mean ± SD. Incident LTCI certification was defined as first certification at Support Level 1 or higher.

Death without LTCI indicates death before any LTCI certification.

HLFC, higher-level functional capacity; LTCI, long-term care insurance; SD, standard deviation.

### 3.2 Association between HLFC and incident LTCI certification

Higher baseline HLFC was associated with a lower cumulative incidence of incident LTCI certification, although the magnitude of association differed across the total and subdomain scores (Table 2). In the unadjusted analyses (Model 1), higher total, instrumental self-maintenance, intellectual activity, and social role scores were each associated with lower cumulative incidence. After adjustment for age group and sex (Model 2), the inverse associations remained for the total score, intellectual activity, and social role, whereas the association for instrumental self-maintenance was attenuated.

**Table 2:** Fine-Gray competing-risk regression for incident LTCI certification.

HLFC measure	Model 1	*p*	Model 2	*p*	Model 3	*p*
	sHR (95% CI)		sHR (95% CI)		sHR (95% CI)	
HLFC total score	0.872 (0.829–0.917)	<0.001	0.919 (0.872–0.967)	0.001	0.941 (0.891–0.994)	0.029
Instrumental self-maintenance	0.797 (0.711–0.893)	<0.001	0.902 (0.801–1.016)	0.089	0.950 (0.840–1.074)	0.414
Intellectual activity	0.718 (0.619–0.833)	<0.001	0.826 (0.708–0.964)	0.015	0.890 (0.758–1.045)	0.153
Social role	0.746 (0.656–0.849)	<0.001	0.772 (0.679–0.877)	<0.001	0.803 (0.702–0.918)	0.001

Each HLFC total or subdomain score was entered separately. Death before LTCI certification was treated as a competing event.

Model 1: unadjusted. Model 2: adjusted for age group and sex.

Model 3: additionally adjusted for living arrangement, physical activity, smoking status, alcohol consumption, and disease status.

sHR, subdistribution hazard ratio; CI, confidence interval; HLFC, higher-level functional capacity; LTCI, long-term care insurance.

In the fully adjusted model (Model 3), which additionally included living arrangement, physical activity, smoking status, alcohol consumption, and disease status, a higher HLFC total score remained associated with a lower cumulative incidence of incident LTCI certification (sHR per 1-point increase, 0.941; 95% CI, 0.891–0.994; *p* = 0.029). A higher social role score was also associated with lower cumulative incidence (sHR, 0.803; 95% CI, 0.702–0.918; *p* = 0.001). In contrast, the estimates for instrumental self-maintenance (sHR, 0.950; 95% CI, 0.840–1.074; *p* = 0.414) and intellectual activity (sHR, 0.890; 95% CI, 0.758–1.045; *p* = 0.153) included the null.

The associations also varied over time. Exposure-by-log(time) interactions were detected for all four measures (all *p* < 0.001), with generally stronger inverse associations earlier in follow-up and attenuation over time (Supplementary Table S3). The prespecified Model 3 sHRs provide whole-follow-up summary estimates and should be interpreted alongside the time-varying results in Supplementary Table S3.

Figure 2 further shows a graded descriptive pattern in cumulative incidence according to baseline social role score. At approximately 8 years, cumulative incidence was 30.5% among participants with scores of 0–2, 21.3% among those with a score of 3, and 16.2% among those with a score of 4.

**Figure 2:**
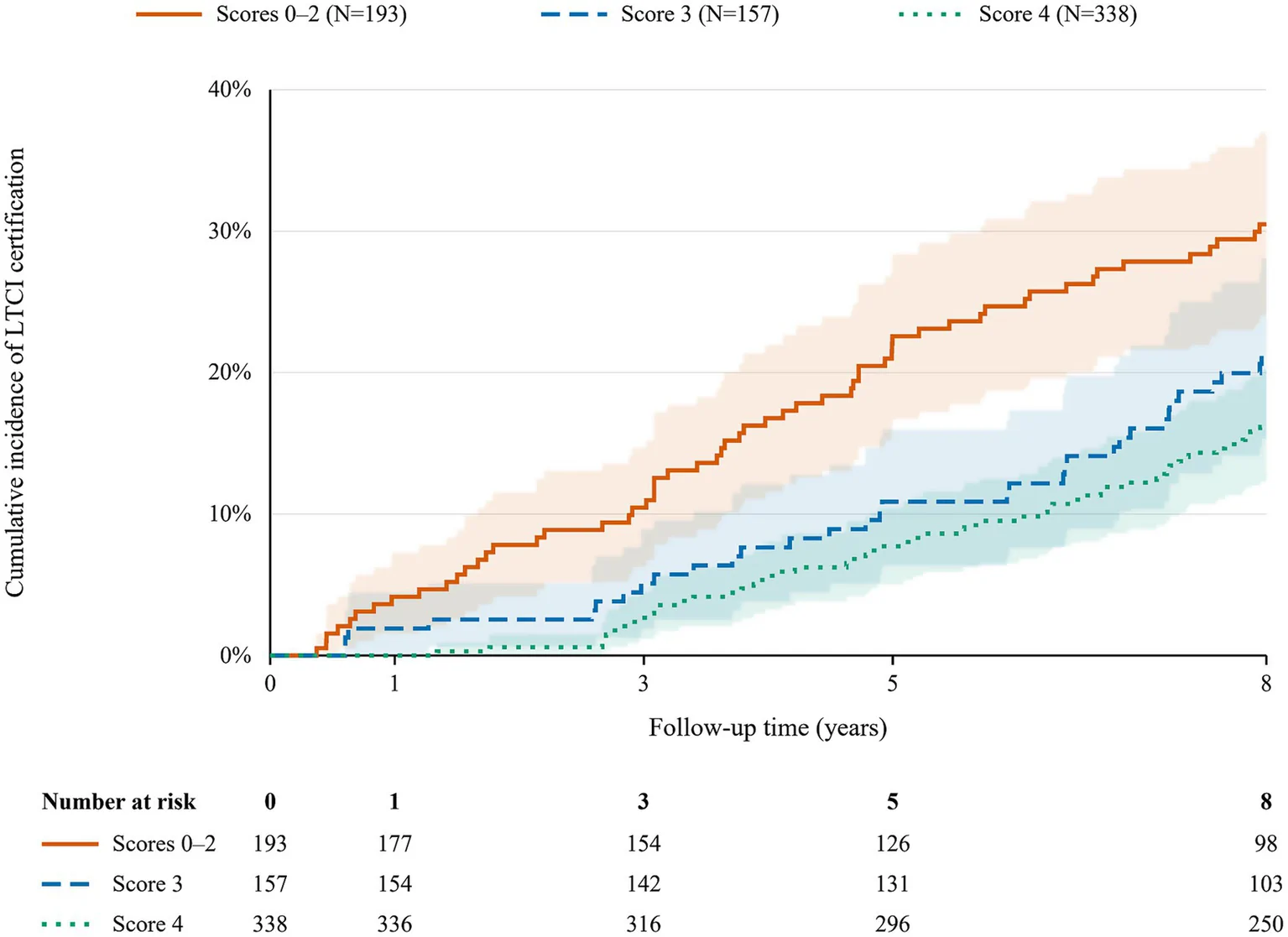
Cumulative incidence of incident LTCI certification by baseline Social Role score group. Scores were grouped as 0–2 (*N* = 193), 3 (*N* = 157), and 4 (*N* = 338), without splitting identical integer scores. Shaded bands indicate pointwise 95% confidence intervals; numbers at risk are shown below the plot.

### 3.3 Sensitivity analyses

Multiple imputation of all 983 eligible participants yielded Model 3 estimates similar in direction and magnitude to the complete-case results. The pooled sHRs were 0.950 (95% CI, 0.906–0.996; *p* = 0.033) for the total score and 0.825 (0.734–0.927; *p* = 0.001) for social role, with corresponding estimates of 0.964 (0.864–1.075; *p* = 0.509) for instrumental self-maintenance and 0.901 (0.785–1.035; *p* = 0.139) for intellectual activity (Supplementary Table S2). Lagged analyses excluding incident LTCI within 6 months or 1 year, as well as exclusion of deaths within the first year, yielded estimates in the same direction and of broadly similar magnitude to the primary analysis (Supplementary Table S5).

Instrumental self-maintenance showed a marked ceiling, with 572 of 688 participants (83.1%) scoring 5/5. Evidence of curvature was detected (quadratic *p* = 0.001). In the dichotomized analysis, scores <5 were associated with a higher cumulative incidence of incident LTCI certification than a score of 5 (sHR, 1.544; 95% CI, 1.038–2.295; *p* = 0.032), supporting a nonlinear or threshold-like pattern (Supplementary Table S4).

On a common 1-SD scale, the Model 3 sHRs were 0.945 (95% CI, 0.825–1.082) for instrumental self-maintenance, 0.891 (0.761–1.044) for intellectual activity, and 0.770 (0.656–0.903) for social role. In the mutually adjusted standardized model, the Social Role estimate was 0.749 (95% CI, 0.623–0.901) per 1-SD higher score. Formal coefficient comparison supported a difference between social role and instrumental self-maintenance (*p* = 0.020), whereas the difference between social role and intellectual activity was not clearly established (*p* = 0.077). Subdomain correlations were moderate (Spearman *ρ* = 0.348–0.432), and variance inflation factors ranged from 1.393 to 1.664 (Supplementary Table S6).

Cause-specific Cox analyses provided complementary endpoint-specific results, with inverse estimates observed for both incident LTCI and death before LTCI certification across several HLFC measures (Supplementary Table S7). Replacing age group with continuous age attenuated the Fine-Gray estimates, with corresponding sHRs of 0.998, 1.094, 1.004, and 0.887 for the total score, instrumental self-maintenance, intellectual activity, and social role, respectively. In models additionally including a quadratic age term, the sHRs were 0.982 (95% CI, 0.922–1.045) for the total score and 0.853 (95% CI, 0.739–0.985) for social role. The estimates therefore varied across age specifications, particularly for the total score (Supplementary Table S8). Among 20 eligible participants who moved out of the municipality, follow-up was censored at moving when no prior event had occurred (Supplementary Table S5).

## 4 Discussion

In this eight-year prospective cohort study of community-dwelling older Japanese adults, higher baseline HLFC total and social role scores were associated with a lower subsequent cumulative incidence of incident LTCI certification after accounting for death as a competing event. Among the HLFC subdomains, Social Role showed an inverse association in the fully adjusted analysis and remained inversely associated with LTCI certification when the three subdomains were entered simultaneously. The overall findings indicate that differences in higher-level functioning at baseline were related to subsequent LTCI certification over long-term follow-up.

The present findings are broadly consistent with previous studies linking poorer higher-level functional capacity and unfavorable HLFC trajectories with disability, mortality, and greater medical or long-term care use in older adults (13–16). Previous research has also examined baseline HLFC scores in relation to subsequent LTCI certification (18). The present study extends this evidence by examining these associations over 8 years and by explicitly treating death before certification as a competing event. This is particularly relevant in older populations because individuals who die before LTCI certification can no longer experience the outcome, and competing-risk analysis therefore provides a direct estimate of cumulative incidence in the presence of mortality (23, 24).

The association observed for social role was one of the main findings of this study. The HLFC social role subdomain includes visiting friends, being asked for advice, visiting sick friends, and initiating conversations with younger people (12). These activities involve interpersonal engagement and participation in everyday social life and overlap with broader concepts of social participation (28). Social activity and social frailty have been associated with incident disability (29, 30). Japanese longitudinal studies have linked social participation with lower risk of incident disability, longer disability-free survival, or lower combined risk of LTC need and mortality (31–35). Other studies have reported associations of socially or cognitively engaging activities with cognitive decline, cognitive impairment, or depressive symptoms in later life (36–40). The present results are therefore consistent with a broader literature linking socially oriented functioning with subsequent health and care-related outcomes.

Several interconnected processes may help explain this association. Previous research has examined walking, cognitive activity, psychological state, and social support as possible pathways linking social participation with incident functional disability (33). These factors may overlap with the broader context represented by the social role items and may help explain why baseline social role remained associated with subsequent LTCI certification after adjustment for the measured demographic, lifestyle, and disease-related factors. In this sense, social role may reflect not only interpersonal engagement itself but also the everyday functional and social environment in which older adults maintain independent community living.

The administrative nature of LTCI certification provides an additional perspective on this association. LTCI certification follows recognition of care needs, application, standardized assessment, and formal certification rather than occurring automatically when health or functioning changes (1, 8, 27). Family members may participate in the assessment, and community general support centers can provide information and assistance with access to LTCI services (2). Social role may therefore be related both to individual higher-level functioning and to the social context in which care needs are recognized and LTCI certification is obtained. This interpretation is particularly relevant when LTCI certification is used as an outcome, because certification reflects both care needs and the administrative process through which that need becomes formally recognized.

The findings across the three HLFC subdomains further illustrate that higher-level functional capacity is multidimensional. On a common 1-SD scale, social role showed the largest inverse point estimate. Formal coefficient comparison supported a difference between social role and instrumental self-maintenance, whereas the difference between social role and intellectual activity was not clearly established. In the mutually adjusted model, social role remained inversely associated with LTCI certification after accounting for the other two subdomains. These findings are compatible with the view that the three HLFC domains capture different aspects of later-life competence and need not show identical associations with subsequent care-need certification.

Instrumental self-maintenance showed a different pattern. Although its continuous per-point association was attenuated after covariate adjustment, most participants scored at the maximum, and additional analyses indicated nonlinearity. Participants scoring below the maximum had a higher cumulative incidence of LTCI certification than those with the maximum score. This pattern may reflect the hierarchical nature of higher-level competence: instrumental self-maintenance represents practical independence in community living, and a departure from full ability may be more informative than small score differences among participants clustered near the upper end of the scale (11, 13, 16, 17). The nonlinear findings therefore provide a different perspective from the continuous analysis and are consistent with the possibility of a threshold-like relationship in this relatively high-functioning population.

Intellectual activity also showed an inverse association in the less-adjusted model, but the estimate was attenuated after full adjustment. Intellectual Activity reflects information-related and cognitively demanding behaviors represented by the TMIG-IC items (12). In a large Japanese cohort, engagement in intellectual and creative activities was associated with a lower risk of cognitive impairment (37). The attenuation after adjustment may reflect shared variation with the measured demographic, lifestyle, and disease-related factors. At the same time, the point estimate remained in the inverse direction, suggesting that intellectual activity and social role may capture partly overlapping but not identical dimensions of higher-level functioning.

The time-varying analyses provided an additional perspective on the primary findings. The inverse associations between baseline HLFC scores and LTCI certification were generally stronger earlier in follow-up and attenuated over time. This pattern suggests that baseline higher-level functioning may be more closely related to LTCI certification occurring nearer to the initial assessment, whereas associations with events occurring many years later are weaker. One possible explanation is that a single baseline assessment becomes less representative of participants’ functional and social circumstances as follow-up lengthens. The prespecified whole-follow-up Fine–Gray estimates should be interpreted as summary associations, while the time-varying analyses show how those associations changed over follow-up.

The study has several strengths. It used a prospective community-based cohort with 8 years of administrative follow-up and explicitly accounted for death before LTCI certification as a competing event. Both the HLFC total score and its component subdomains were examined, allowing the overall measure of higher-level functional capacity to be considered alongside the different dimensions represented by instrumental self-maintenance, intellectual activity, and social role. The additional analyses addressed missing data, early events, nonlinearity, standardized domain comparisons, mutually adjusted domain models, and alternative time-to-event specifications. Multiple-imputation and lagged-event analyses yielded estimates that were broadly similar in direction and magnitude to the primary complete-case findings, and cause-specific Cox models provided complementary endpoint-specific information.

Several considerations should be taken into account when interpreting the findings. HLFC was assessed only at baseline, so the study describes baseline differences in higher-level functioning rather than within-person changes during follow-up. The primary analysis was based on complete cases, although multiple-imputation analyses produced broadly similar estimates. Associations varied over follow-up, and age specification influenced the estimated associations. Modeling age continuously attenuated the estimates, particularly for the total score, while quadratic-age models produced different estimates for both the total score and social role. Dichotomizing age as 65–74 and ≥75 years may therefore not fully capture the relation between age and LTCI certification and may leave residual confounding by age. In addition, the available covariates did not fully capture physical function, cognition, depressive symptoms, frailty, socioeconomic circumstances, or disease severity, and residual confounding therefore remains possible. Generalizability may also be limited because the study was conducted in a rural village in Aichi Prefecture, Japan, which may differ from other settings in population composition, socioeconomic context, social resources, service access, and the local LTCI-related environment.

## 5 Conclusion

Baseline higher-level functional capacity, particularly social role, was associated with subsequent LTCI certification among community-dwelling older Japanese adults. The association for the overall HLFC score was substantially attenuated under alternative age adjustments, and the magnitude of the Social Role association also varied. Social role captures interpersonal and community-related functioning in everyday life, an aspect of later-life competence that may be relevant to understanding subsequent LTCI certification.

## Data Availability

The datasets analyzed in this study are not publicly available due to privacy and data-sharing restrictions associated with municipal administrative data. Requests to access the datasets may be directed to the corresponding author and are subject to approval by the relevant data provider and institutional requirements.
