# Supplementary material for: Higher-level functional capacity and incident long-term care insurance certification: a competing risks analysis
Source: Front Public Health. 2026 Oct 6;14:1950765. doi: 10.3389/fpubh.2026.1950765 (PMC13680145; doi:10.3389/fpubh.2026.1950765)
Supplement: Supplementary file 1 [file Table_1.DOCX]

Supplementary Material

# Supplementary Tables

**Table S1. Missing data and baseline characteristics of participants included in and excluded from the complete-case analysis**

**Panel A. Variable-specific missingness among 983 eligible participants**

| **Variable** | **Missing, n (%)** | **Available, n (%)** |
| --- | --- | --- |
| Follow-up time | 0 (0.0) | 983 (100.0) |
| Outcome status | 0 (0.0) | 983 (100.0) |
| HLFC total score | 175 (17.8) | 808 (82.2) |
| Instrumental Self-Maintenance | 112 (11.4) | 871 (88.6) |
| Intellectual Activity | 114 (11.6) | 869 (88.4) |
| Social Role | 113 (11.5) | 870 (88.5) |
| Age group | 0 (0.0) | 983 (100.0) |
| Sex | 0 (0.0) | 983 (100.0) |
| Living arrangement | 131 (13.3) | 852 (86.7) |
| Physical activity | 107 (10.9) | 876 (89.1) |
| Smoking status | 49 (5.0) | 934 (95.0) |
| Alcohol consumption | 43 (4.4) | 940 (95.6) |
| Disease status | 96 (9.8) | 887 (90.2) |

**Panel B. Overlap of missing primary-analysis variables**

| **Missingness category** | **Participants, n** | **Participants, %** |
| --- | --- | --- |
| Complete | 688 | 70.0 |
| Exactly 1 variable | 149 | 15.2 |
| Exactly 2 variables | 76 | 7.7 |
| 3 or more variables | 70 | 7.1 |

**Panel C. Continuous characteristics**

| **Characteristic** | **Included (n=688), mean±SD** | **Excluded,**  **n available** | **Excluded, mean±SD** | **SMD** |
| --- | --- | --- | --- | --- |
| Age, years | 73.44±6.88 | 295 | 75.49±7.82 | -0.279 |
| HLFC total score | 10.93±2.64 | 120 | 10.84±2.77 | 0.032 |
| Instrumental Self-Maintenance | 4.60±1.10 | 183 | 4.68±1.02 | -0.076 |
| Intellectual Activity | 3.30±0.98 | 181 | 3.23±1.06 | 0.064 |
| Social Role | 3.03±1.19 | 182 | 2.93±1.19 | 0.079 |

**Panel D. Categorical characteristics**

| **Characteristic** | **Category** | **Included,**  **n (%)** | **Excluded,**  **n available** | **Excluded,**  **n (%)** | **SMD** |
| --- | --- | --- | --- | --- | --- |
| Age group | 65–74 | 432 (62.8) | 295 | 155 (52.5) | 0.209 |
|  | ≥75 | 256 (37.2) | 295 | 140 (47.5) | -0.209 |
| Sex | Female | 350 (50.9) | 295 | 174 (59.0) | -0.164 |
|  | Male | 338 (49.1) | 295 | 121 (41.0) | 0.164 |
| Living arrangement | With others | 647 (94.0) | 164 | 152 (92.7) | 0.055 |
|  | Alone | 41 (6.0) | 164 | 12 (7.3) | -0.055 |
| Physical activity | Inactive | 273 (39.7) | 188 | 87 (46.3) | -0.134 |
|  | Active | 415 (60.3) | 188 | 101 (53.7) | 0.134 |
| Smoking status | Never | 425 (61.8) | 246 | 163 (66.3) | -0.094 |
|  | Former | 180 (26.2) | 246 | 61 (24.8) | 0.031 |
|  | Current | 83 (12.1) | 246 | 22 (8.9) | 0.102 |
| Alcohol consumption | Daily | 263 (38.2) | 252 | 63 (25.0) | 0.287 |
|  | Non-daily | 425 (61.8) | 252 | 189 (75.0) | -0.287 |
| Disease status | Without disease | 105 (15.3) | 199 | 30 (15.1) | 0.005 |
|  | Having disease | 583 (84.7) | 199 | 169 (84.9) | -0.005 |

*Note.* HLFC, higher-level functional capacity; LTCI, long-term care insurance; SMD, standardized mean difference. Values in Panel C are presented as mean±SD. “Excluded, n available” indicates the number of excluded participants with non-missing data for the corresponding variable. Percentages for excluded participants in Panel D were calculated using the number with available data for each variable. Absolute SMDs <0.10 were considered little, 0.10–<0.20 moderate, and ≥0.20 notable.

**Table S2. Multiple-imputation sensitivity analysis of Fine-Gray models for incident LTCI certification**

**Panel A. Pooled multiple-imputation results**

| **Variable** | **Model** | **sHR (95% CI)** | ***p*** | **FMI** |
| --- | --- | --- | --- | --- |
| HLFC total score | Model 1 | 0.892 (0.854–0.931) | <0.001 | 0.133 |
|  | Model 2 | 0.936 (0.895–0.978) | 0.004 | 0.125 |
|  | Model 3 | 0.950 (0.906–0.996) | 0.033 | 0.152 |
| Instrumental Self-Maintenance | Model 1 | 0.833 (0.754–0.920) | <0.001 | 0.158 |
|  | Model 2 | 0.933 (0.840–1.037) | 0.199 | 0.155 |
|  | Model 3 | 0.964 (0.864–1.075) | 0.509 | 0.178 |
| Intellectual Activity | Model 1 | 0.755 (0.667–0.854) | <0.001 | 0.122 |
|  | Model 2 | 0.861 (0.756–0.981) | 0.024 | 0.129 |
|  | Model 3 | 0.901 (0.785–1.035) | 0.139 | 0.158 |
| Social Role | Model 1 | 0.768 (0.689–0.857) | <0.001 | 0.097 |
|  | Model 2 | 0.804 (0.719–0.898) | <0.001 | 0.104 |
|  | Model 3 | 0.825 (0.734–0.927) | 0.001 | 0.114 |

*Note.* N=983; 222 incident LTCI certifications and 115 deaths before certification. Model 1 was unadjusted; Model 2 was adjusted for age group and sex; Model 3 was additionally adjusted for living arrangement, physical activity, smoking status, alcohol consumption, and disease status. Death before LTCI certification was treated as a competing event. FMI, fraction of missing information; HLFC, higher-level functional capacity; LTCI, long-term care insurance; sHR, subdistribution hazard ratio; CI, confidence interval.

**Panel B. Complete-case versus multiple-imputation Model 3 estimates**

| **Variable** | **sHR (95% CI)** | ***p*** | **MI, sHR (95% CI)** | ***p*** |
| --- | --- | --- | --- | --- |
| HLFC total score | 0.941 (0.891–0.994) | 0.029 | 0.950 (0.906–0.996) | 0.033 |
| Instrumental Self-Maintenance | 0.950 (0.840–1.074) | 0.414 | 0.964 (0.864–1.075) | 0.509 |
| Intellectual Activity | 0.890 (0.758–1.045) | 0.153 | 0.901 (0.785–1.035) | 0.139 |
| Social Role | 0.803 (0.702–0.918) | 0.001 | 0.825 (0.734–0.927) | 0.001 |

*Note.* Model 3 adjusted for age group, sex, living arrangement, physical activity, smoking, alcohol consumption, and disease status. Complete-case: N=688 (145 LTCI certifications; 79 deaths); MI: N=983 (222 LTCI certifications; 115 deaths). CI, confidence interval; HLFC, higher-level functional capacity; LTCI, long-term care insurance; MI, multiple imputation; sHR, subdistribution hazard ratio.

**Table S3. Proportional subdistribution hazards diagnostics and time-specific Fine-Gray estimates**

**Panel A. Exposure-by-log (time) diagnostics in Model 3**

| **Variable** | **Exposure × log(time) β (95% CI)** | **exp(β) (95% CI)** | ***p*** |
| --- | --- | --- | --- |
| HLFC total score | 0.135 (0.088–0.183) | 1.145 (1.092–1.200) | <0.001 |
| Instrumental Self-Maintenance | 0.288 (0.178–0.398) | 1.334 (1.195–1.489) | <0.001 |
| Intellectual Activity | 0.364 (0.204–0.525) | 1.440 (1.226–1.691) | <0.001 |
| Social Role | 0.320 (0.156–0.484) | 1.377 (1.168–1.623) | <0.001 |

**Panel B. Time-specific Model 3 sHR estimates**

| **Variable** | **1 year; sHR (95% CI)** | **3 years; sHR (95% CI)** | **5 years; sHR (95% CI)** | **8 years; sHR (95% CI)** |
| --- | --- | --- | --- | --- |
| HLFC total score | 0.803  (0.746–0.864) | 0.932  (0.883–0.983) | 0.998  (0.940–1.060) | 1.064  (0.990–1.144) |
| Instrumental Self-Maintenance | 0.693  (0.589–0.816) | 0.951  (0.840–1.077) | 1.102  (0.956–1.270) | 1.262  (1.061–1.501) |
| Intellectual Activity | 0.569  (0.445–0.727) | 0.849  (0.722–0.999) | 1.023  (0.855–1.223) | 1.213  (0.973–1.514) |
| Social Role | 0.533  (0.418–0.680) | 0.757  (0.661–0.868) | 0.892  (0.770–1.034) | 1.036  (0.855–1.257) |

*Note.* N=688 (145 incident LTCI certifications; 79 competing deaths). Model 3 was adjusted for age group, sex, living arrangement, physical activity, smoking status, alcohol consumption, and disease status. Death before LTCI certification was treated as a competing event. A significant exposure × log(time) interaction indicates non-proportional subdistribution hazards. Numbers at risk at 1, 3, 5, and approximately 8 years were 667, 612, 553, and 451, respectively. CI, confidence interval; HLFC, higher-level functional capacity; LTCI, long-term care insurance; sHR, subdistribution hazard ratio.

**Table S4. HLFC score distributions, ceiling effects, nonlinear analyses, and descriptive Social Role cumulative incidence**

**Panel A. Score distributions**

| **Variable** | **Mean±SD** | **Median** | **Q1–Q3** | **Range** | **Maximum, n (%)** |
| --- | --- | --- | --- | --- | --- |
| HLFC total score | 10.93±2.64 | 12 | 10–13 | 0–13 | 234 (34.0) |
| Instrumental Self-Maintenance | 4.60±1.10 | 5 | 5–5 | 0–5 | 572 (83.1) |
| Intellectual Activity | 3.30±0.98 | 4 | 3–4 | 0–4 | 388 (56.4) |
| Social Role | 3.03±1.19 | 3 | 2–4 | 0–4 | 338 (49.1) |

**Panel B. Quadratic curvature screen in Model 3**

| **Variable** | **Quadratic β (95% CI)** | ***p*** |
| --- | --- | --- |
| HLFC total score | -0.013 (-0.028–0.001) | 0.066 |
| Instrumental Self-Maintenance | -0.176 (-0.281–-0.070) | 0.001 |
| Intellectual Activity | -0.015 (-0.135–0.104) | 0.800 |
| Social Role | 0.026 (-0.093–0.145) | 0.671 |

**Panel C. Categorical Instrumental Self-Maintenance analyses in Model 3**

| **Analysis** | **N; LTCI/deaths** | **sHR (95% CI)** | ***p*** |
| --- | --- | --- | --- |
| **<5 vs 5** |  |  |  |
| <5 | 116; 41/26 | 1.544 (1.038–2.295) | 0.032 |
| 5 | 572; 104/53 | Reference | — |
| **Three-level analysis** |  |  |  |
| 0–3 | 58; 23/15 | 1.618 (0.953–2.747) | 0.075 |
| 4 | 58; 18/11 | 1.461 (0.860–2.482) | 0.161 |
| 5 | 572; 104/53 | Reference | — |

**Panel D. Cumulative incidence by baseline Social Role score group**

| **Group** | **N** | **1 year, %**  **(95% CI)** | **3 years, % (95% CI)** | **5 years, %**  **(95% CI)** | **~8 years, %**  **(95% CI)** |
| --- | --- | --- | --- | --- | --- |
| Scores 0–2 | 193 | 4.2 (1.6–6.8) | 10.5 (6.3–15.2) | 22.6 (16.7–28.5) | 30.5 (24.2–37.3) |
| Score 3 | 157 | 1.9 (0.0–4.5) | 5.1 (1.9–8.3) | 10.9 (6.4–15.4) | 21.3 (14.9–28.0) |
| Score 4 | 338 | 0.0 (0.0–0.0) | 2.7 (1.2–4.7) | 7.7 (4.8–10.7) | 16.2 (12.2–20.4) |

**Panel E. Numbers at risk for Figure 2**

| **Group** | **0 year** | **1 year** | **3 years** | **5 years** | **~8 years** |
| --- | --- | --- | --- | --- | --- |
| Scores 0–2 | 193 | 177 | 154 | 126 | 98 |
| Score 3 | 157 | 154 | 142 | 131 | 103 |
| Score 4 | 338 | 336 | 316 | 296 | 250 |

*Note.* Model 3 was adjusted for age group, sex, living arrangement, physical activity, smoking status, alcohol consumption, and disease status. Quadratic terms were based on centered scores. Instrumental Self-Maintenance and Social Role groups preserved identical integer scores. The global p value for the three-level Instrumental Self-Maintenance analysis was 0.100. Social Role cumulative-incidence confidence intervals were pointwise bootstrap 95% CIs; no formal between-group test was performed. CI, confidence interval; HLFC, higher-level functional capacity; LTCI, long-term care insurance; sHR, subdistribution hazard ratio.

**Table S5. Lagged incident-LTCI, early-death exclusion, and censoring sensitivity summaries**

**Panel A. Sensitivity-analysis cohort flow**

| **Analysis** | **Excluded, n** | **Analysis N** | **LTCI/deaths/censored, n** |
| --- | --- | --- | --- |
| Primary complete-case Model 3 | 0 | 688 | 145/79/464 |
| Exclude LTCI within 6 months | 3 | 685 | 142/79/464 |
| Exclude LTCI within 1 year | 11 | 677 | 134/79/464 |
| Exclude deaths within 1 year | 8 | 680 | 145/71/464 |

**Panel B. Model 3 estimates**

| **Variable** | **LTCI ≤183 days, sHR (95% CI); *p*** | **LTCI ≤365 days, sHR (95% CI); *p*** | **Deaths ≤365 days, sHR (95% CI); *p*** |
| --- | --- | --- | --- |
| HLFC total score | 0.950 (0.899–1.004); *p*=0.071 | 0.963 (0.908–1.020); *p*=0.200 | 0.935 (0.884–0.989); *p*=0.020 |
| Instrumental Self-Maintenance | 0.966 (0.851–1.098); *p*=0.597 | 0.999 (0.875–1.140); *p*=0.987 | 0.939 (0.828–1.066); *p*=0.331 |
| Intellectual Activity | 0.922 (0.785–1.084); *p*=0.326 | 0.937 (0.790–1.110); *p*=0.452 | 0.882 (0.750–1.037); *p*=0.128 |
| Social Role | 0.816 (0.712–0.934); *p*=0.003 | 0.842 (0.730–0.970); *p*=0.017 | 0.795 (0.694–0.910);  *p*<0.001 |

**Panel C. Out-migration and censoring audit**

| **Participant** | **N** | **Moved,**  **n (%)** | **Censored at move,**  **n** | **LTCI before move,**  **n** | **Post-move follow-up/event, n** |
| --- | --- | --- | --- | --- | --- |
| Baseline records | 1118 | 21 (1.9) | 15 | 6 | 0 |
| Eligible participants | 983 | 20 (2.0) | 15 | 5 | 0 |
| Complete cases | 688 | 17 (2.5) | 13 | 4 | 0 |

*Note.* Model 3 adjusted for age group, sex, living arrangement, physical activity, smoking, alcohol consumption, and disease status; death before LTCI certification was treated as a competing event. Event-free movers were censored at the moving date. CI, confidence interval; HLFC, higher-level functional capacity; LTCI, long-term care insurance; sHR, subdistribution hazard ratio.

**Table S6. Standardized and mutually adjusted HLFC subdomain analyses, pairwise comparisons, correlations, and multicollinearity**

**Panel A. Separate-subdomain Model 3 estimates per 1 SD higher baseline score**

| **Subdomain** | **SD** | **sHR (95% CI)** | ***p*** |
| --- | --- | --- | --- |
| Instrumental Self-Maintenance | 1.104 | 0.945 (0.825–1.082) | 0.414 |
| Intellectual Activity | 0.983 | 0.891 (0.761–1.044) | 0.153 |
| Social Role | 1.191 | 0.770 (0.656–0.903) | 0.001 |

**Panel B. Mutually adjusted Model 3 estimates**

| **Subdomain** | **Per 1 point, sHR (95% CI); *p*** | **Per 1 SD, sHR (95% CI); *p*** |
| --- | --- | --- |
| Instrumental Self-Maintenance | 1.062 (0.902–1.250); 0.470 | 1.069 (0.893–1.279); 0.470 |
| Intellectual Activity | 0.979 (0.800–1.198); 0.836 | 0.979 (0.803–1.195); 0.836 |
| Social Role | 0.785 (0.672–0.917); 0.002 | 0.749 (0.623–0.901); 0.002 |

**Panel C. Pairwise comparisons in the standardized mutually adjusted model**

| **Comparison** | **β difference (95% CI)** | ***p*** |
| --- | --- | --- |
| Social Role versus Instrumental Self-Maintenance | -0.355 (-0.653 to -0.056) | 0.020 |
| Social Role versus Intellectual Activity | -0.268 (-0.564 to 0.029) | 0.077 |
| Instrumental Self-Maintenance versus Intellectual Activity | 0.087 (-0.237 to 0.412) | 0.598 |

**Panel D. Spearman rank-correlation matrix**

| **Subdomain** | **Instrumental Self-Maintenance** | **Intellectual Activity** | **Social Role** |
| --- | --- | --- | --- |
| Instrumental Self-Maintenance | 1.000 | 0.386 | 0.348 |
| Intellectual Activity | 0.386 | 1.000 | 0.432 |
| Social Role | 0.348 | 0.432 | 1.000 |

**Panel E. Multicollinearity diagnostics**

| **Subdomain** | **Tolerance** | **VIF** |
| --- | --- | --- |
| Instrumental Self-Maintenance | 0.662 | 1.511 |
| Intellectual Activity | 0.601 | 1.664 |
| Social Role | 0.718 | 1.393 |

*Note.* N=688; 145 incident LTCI certifications and 79 competing deaths. Model 3 adjusted for age group, sex, living arrangement, physical activity, smoking status, alcohol consumption, and disease status; death before LTCI certification was treated as a competing event. All three subdomains were entered simultaneously in Panel B. Pairwise comparisons in Panel C were covariance-based Wald tests of standardized coefficients from the mutually adjusted model. CI, confidence interval; HLFC, higher-level functional capacity; LTCI, long-term care insurance; SD, standard deviation; sHR, subdistribution hazard ratio; VIF, variance inflation factor.

**Table S7. Cause-specific Cox analyses for incident LTCI certification and death before LTCI certification**

**Panel A. Constant cause-specific Cox Model 3 estimates**

| **Endpoint** | **Variable** | **HR (95% CI)** | ***p*** | **Exposure PH *p*** | **Global PH *p*** |
| --- | --- | --- | --- | --- | --- |
| Incident LTCI certification | HLFC total score | 0.913 (0.866–0.962) | <0.001 | <0.001 | 0.003 |
|  | Instrumental Self-Maintenance | 0.904 (0.802–1.019) | 0.099 | <0.001 | 0.002 |
|  | Intellectual Activity | 0.840 (0.720–0.979) | 0.026 | <0.001 | 0.005 |
|  | Social Role | 0.753 (0.663–0.856) | <0.001 | 0.008 | 0.012 |
| Death before LTCI certification | HLFC total score | 0.825 (0.767–0.886) | <0.001 | 0.923 | 0.547 |
|  | Instrumental Self-Maintenance | 0.705 (0.610–0.816) | <0.001 | 0.811 | 0.663 |
|  | Intellectual Activity | 0.679 (0.548–0.843) | <0.001 | 0.878 | 0.681 |
|  | Social Role | 0.712 (0.600–0.846) | <0.001 | 0.572 | 0.569 |

**Panel B. Time-specific cause-specific HRs for incident LTCI certification**

| **Variable** | **1 year,** **HR (95% CI)** | **3 years, HR**  **(95% CI)** | **5 years, HR (95% CI)** | **~8 years, HR (95% CI)** | **Time-interaction** ***p*** |
| --- | --- | --- | --- | --- | --- |
| HLFC total score | 0.798  (0.731–0.871) | 0.907  (0.858–0.959) | 0.963  (0.902–1.028) | 1.017  (0.934–1.108) | <0.001 |
| Instrumental Self-Maintenance | 0.688  (0.573–0.825) | 0.915  (0.802–1.045) | 1.046  (0.886–1.234) | 1.182  (0.953–1.465) | <0.001 |
| Intellectual Activity | 0.566  (0.431–0.743) | 0.808  (0.689–0.949) | 0.954  (0.796–1.144) | 1.111  (0.876–1.409) | <0.001 |
| Social Role | 0.534  (0.409–0.696) | 0.718 (0.627–0.823) | 0.824  (0.712–0.954) | 0.936  (0.768–1.141) | 0.003 |

*Note.* N=688. Model 3 adjusted for age group, sex, living arrangement, physical activity, smoking status, alcohol consumption, and disease status. For the incident-LTCI endpoint, death before LTCI certification was censored; for the death endpoint, incident LTCI certification was censored. Exposure PH and global PH p values assess the proportional-hazards assumption. Time-interaction p values in Panel B assess exposure × log(time). Numbers at risk at 1, 3, 5, and approximately 8 years were 667, 612, 553, and 451, respectively. CI, confidence interval; HLFC, higher-level functional capacity; HR, hazard ratio; LTCI, long-term care insurance; PH, proportional hazards.

**Table S8. Fine-Gray sensitivity analyses using continuous and quadratic age**

**Panel A. Continuous age replacing age group in Model 3**

| **Variable** | **Continuous age**  **sHR (95% CI)** | ***p*** | **Primary age group**  **sHR (95% CI)** |
| --- | --- | --- | --- |
| HLFC total score | 0.998 (0.936–1.063) | 0.942 | 0.941 (0.891–0.994) |
| Instrumental Self-Maintenance | 1.094 (0.942–1.272) | 0.239 | 0.950 (0.840–1.074) |
| Intellectual Activity | 1.004 (0.839–1.202) | 0.966 | 0.890 (0.758–1.045) |
| Social Role | 0.887 (0.768–1.024) | 0.102 | 0.803 (0.702–0.918) |

**Panel B. Quadratic-age diagnostic**

| **Variable** | **Quadratic-age *p*** | **Exposure sHR (95% CI)** | ***p*** |
| --- | --- | --- | --- |
| HLFC total score | 0.048 | 0.982 (0.922–1.045) | 0.560 |
| Instrumental Self-Maintenance | 0.098 | 1.061 (0.916–1.230) | 0.428 |
| Intellectual Activity | 0.055 | 0.979 (0.823–1.165) | 0.810 |
| Social Role | 0.024 | 0.853 (0.739–0.985) | 0.030 |

*Note.* Model 3 adjusted for sex, living arrangement, physical activity, smoking status, alcohol consumption, and disease status, with age specified as shown. In Panel A, continuous age replaced the primary age-group variable (65–74 vs ≥75 years). Panel B additionally included centered continuous age and a quadratic age term as a diagnostic sensitivity analysis; these models did not replace the prespecified age-group-adjusted primary model. CI, confidence interval; HLFC, higher-level functional capacity; sHR, subdistribution hazard ratio.

**Table S9. Items of the Tokyo Metropolitan Institute of Gerontology Index of Competence and formal subdomains**

| **Subdomain** | **Item** | **Item wording** |
| --- | --- | --- |
| Instrumental Self-Maintenance | 1 | Can you use public transportation (bus or train) by yourself? |
|  | 2 | Are you able to shop for daily necessities? |
|  | 3 | Are you able to prepare meals by yourself? |
|  | 4 | Are you able to pay bills? |
|  | 5 | Can you handle your own banking? |
| Intellectual Activity | 6 | Are you able to fill out forms for your pension? |
|  | 7 | Do you read newspapers? |
|  | 8 | Do you read books or magazines? |
|  | 9 | Are you interested in news stories or programs dealing with health? |
| Social Role | 10 | Do you visit friends' homes? |
|  | 11 | Are you sometimes called on for advice? |
|  | 12 | Are you able to visit sick friends? |
|  | 13 | Do you sometimes initiate conversations with young people? |

*Note.* The TMIG-IC consists of 13 yes/no items grouped into Instrumental Self-Maintenance (items 1–5), Intellectual Activity (items 6–9), and Social Role (items 10–13), following the formal subdomain structure reported by Koyano et al. (12). Each item is scored 1 for “yes” and 0 for “no.” Possible scores range from 0 to 13 for the total score, 0 to 5 for Instrumental Self-Maintenance, and 0 to 4 for Intellectual Activity and Social Role.
